# Matrix metalloproteinase-7 facilitates immune access to the CNS in experimental autoimmune encephalomyelitis

**DOI:** 10.1186/1471-2202-10-17

**Published:** 2009-03-06

**Authors:** Lillian A Buhler, Ramsey Samara, Esther Guzman, Carole L Wilson, Liljana Krizanac-Bengez, Damir Janigro, Douglas W Ethell

**Affiliations:** 1 Division of Biomedical Sciences, University of California Riverside, 900 University Avenue, Riverside, CA 92521-0121, USA; 2 Biochemistry and Molecular Biology Graduate Program, UCR, Riverside, CA 92521, USA; 3 Neuroscience Graduate Program, UCR, Riverside, CA 92521, USA; 4 Department of Pathology, University of Washington School of Medicine, 300 9th Avenue, Seattle, WA 98104, USA; 5 Cerebrovascular Research, Cleveland Clinic Foundation NB20, Neurosurgery, 9500 Euclid Avenue, Cleveland, OH 44195, USA

## Abstract

**Background:**

Metalloproteinase inhibitors can protect mice against experimental autoimmune encephalomyelitis (EAE), an animal model for multiple sclerosis (MS). Matrix metalloproteinase-9 (MMP-9) has been implicated, but it is not clear if other MMPs are also involved, including matrilysin/MMP-7 – an enzyme capable of cleaving proteins that are essential for blood brain barrier integrity and immune suppression.

**Results:**

Here we report that MMP-7-deficient (*mmp7*^-/-^) mice on the C57Bl/6 background are resistant to EAE induced by myelin oligodendrocyte glycoprotein (MOG). Brain sections from MOG-primed *mmp7*^-/-^mice did not show signs of immune cell infiltration of the CNS, but MOG-primed wild-type mice showed extensive vascular cuffing and mononuclear cell infiltration 15 days after vaccination. At the peak of EAE wild-type mice had MMP-7 immuno-reactive cells in vascular cuffs that also expressed the macrophage markers Iba-1 and Gr-1, as well as tomato lectin. MOG-specific proliferation of splenocytes, lymphocytes, CD4^+ ^and CD8^+ ^cells were reduced in cells isolated from MOG-primed *mmp7*^-/- ^mice, compared with MOG-primed wild-type mice. However, the adoptive transfer of splenocytes and lymphocytes from MOG-primed *mmp7*^-/- ^mice induced EAE in naïve wild-type recipients, but not naïve *mmp7*^-/- ^recipients. Finally, we found that recombinant MMP-7 increased permeability between endothelial cells in an *in vitro *blood-brain barrier model.

**Conclusion:**

Our findings suggest that MMP-7 may facilitate immune cell access or re-stimulation in perivascular areas, which are critical events in EAE and multiple sclerosis, and provide a new therapeutic target to treat this disorder.

## Background

Multiple sclerosis (MS) is an autoimmune disorder marked by the infiltration of pathogenic T cells into the central nervous system (CNS) that cause inflammation and oligodendrocyte cell death. In an animal model of MS, called experimental autoimmune encephalomyelitis (EAE), vaccination with CNS-myelin-derived peptides triggers the expansion of oligodendrocyte-specific T cells and a pathological profile that includes CNS inflammation, demyelination, and paralysis. Transmigration of pathogenic T cells across the blood-brain barrier (BBB) is facilitated by the expression of cell adhesion molecules and proteinases that degrade the ECM [1]. The discovery that EAE can be prevented by broad spectrum metalloproteinase inhibitors implicated this large family of enzymes in disease progression [1-5] and has led to recent clinical trials [6]. Matrix metalloproteinases (MMPs) are extracellular enzymes that can cleave ECM and non-matrix proteins, including laminin, collagen, cytokines, other proteinases, and the ectodomains of several membrane proteins. MMPs are usually secreted as pro-enzymes that can be cleavage-activated by plasminogen activators, trypsin, other MMPs, and oxidation. Elevated levels of MMP-2, MMP-7 and MMP-9 have been reported in human MS patients, and in brain and spinal cord extracts from EAE-induced rodents [7-17]. In a delayed-type hypersensitivity model for MS, MMP-7 was found to be the most up-regulated MMP, compared with MMP-2,3,8,9,10,11,12,13,14,15 and 16 [11].

Within tissues, MMPs usually reside in extracellular spaces as inactive proforms, and factors that activate even a small proportion of those MMPs have significant biological effects. Therefore, determining which factors contribute to MMP activity in MS will be critical to understanding the role(s) these enzymes play in this disorder. Cerebrospinal fluid levels of MMP-9 activity are elevated in MS patients and in rodent models of EAE [18], and young MMP-9 knockout mice (4 weeks) are resistant to EAE [19]. MMP-2 plays a critical role in angiogenesis and vascular remodeling [20]. Although MMP-2 expression does not increase in MS or EAE, MMP-2 activation may contribute to localized permeabilization of the cerebrovasculature. MMP-2 and MMP-9 are structurally similar gelatinases that can each be activated by MMP-7 [21]. MMP-7 can also cleave many EAE-relevant substrates, including laminin, type IV collagen [22], β4-integrin [23], VE-cadherin [24], E-cadherin [25-27] and the immune suppressor Fas ligand (FasL) [28]. Further, MMP-7 has been reported as necessary for the trans-epithelial efflux of immune cells in bleomycin-treated lungs [29], which is similar to the extravasation that immune cells must make in EAE and MS.

Myelin-specific T cells can be detected in the blood of MS patients and EAE-induced mice even during periods of remission, when they no longer persist in the CNS. Tight junctions between microvascular endothelial cells within the brain prevent the direct entry of macromolecules and blood-borne cells, forming the BBB. Compromise of BBB integrity facilitates immune cell access to the CNS and is essential for MS and EAE. For example, MRI detection of gadolinium accumulation in the brain lesions of MS patients is an indicator of compromised BBB integrity and a reliable predictor of pending disease activity. Factors that affect the cell-to-cell contacts of cerebrovascular endothelial cells, or their viability, can reduce BBB integrity and increase immune cell access to the CNS. VE-cadherin is an important component of tight junctions between endothelial cells and is also a substrate for cleavage by both MMP-7 [24] and MMP-9 [30]. The two layers or ECM that surround the cerebrovasculature contain laminin and type IV collagen, which are cleaved by MMP-7 [22], as well as collagens and elastins are cleaved by MMP-9 [31].

In addition to the BBB, immune cells actions are restricted within the CNS action by the expression of cell death ligands CD95L/Fas ligand/FasL and TRAIL that can trigger apoptosis in activated T cells and myeloid cells. FasL is a potent inducer of apoptosis in activated T cells [32] and its expression is elevated in response to injuries that compromise BBB integrity [15,33-35]. Facial nerve damage locally increases FasL expression and T cell apoptosis around the facial nucleus in EAE-induced mice [36]. The availability of FasL is closely regulated at the transcriptional and post-translational levels, which includes proteolytic cleavage of its receptor binding region by MMP-3 [37] and MMP-7 [28,38]. While membrane-bound FasL has potent pro-apoptotic activity for activated T cells, cleaved or soluble FasL (sFasL) shows differential effects, depending on the disease model and specific cell types [39,40]. Moreover, sFasL has a chemotaxic effect on mononuclear cells, which can be blocked by anti-FasL antibodies [41].

Here, we used MMP-7-deficient (*mmp7*^-/-^) mice to investigate the role of this proteinase in EAE. Wild-type (*wt*) and *mmp7*^-/- ^mice were compared for clinical and immunological responses to an encephalitogenic fragment of myelin oligodendrocyte protein peptide (MOG_35–55_). Localization of MMP-7 expression in the CNS was examined during EAE in MOG-primed mice at the peak of EAE. MOG-specific proliferation of splenocytes, lymphocytes, CD4^+^, and CD8^+ ^T cells were compared in both strains of mice. We also used adoptive transfer to assess the encephalitogenic potential of T cells from *wt *and *mmp7*^-/- ^mice to induce EAE in recipients of both strains. Finally, we tested the effects of active MMP-7 on endothelial cell connectivity using an established *in vitro *model of the BBB.

## Results

### *mmp7*^-/- ^Mice Are Resistant to EAE

**Figure 1:**
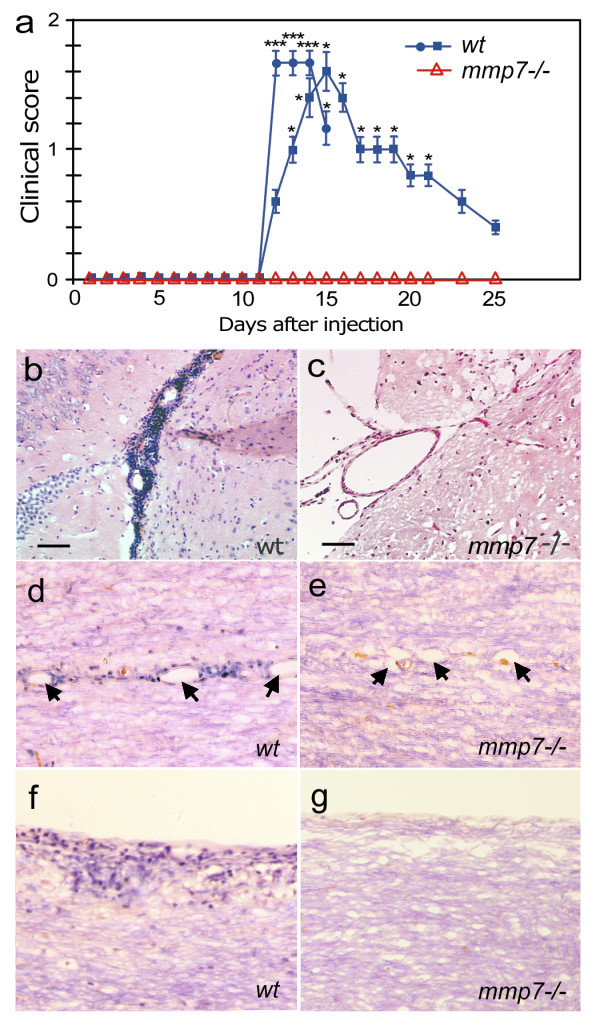
**Wild-type and *mmp7*^-/- ^mice show respond differently to vaccination with MOG_35–55_**. (a) Mean clinical EAE scores from *wt *(n = 12) and *mmp7*^-/- ^(n = 9) mice injected with MOG_35–55_: graphed values are from two separate experiments. The first cadre of mice were sacrificed at day 15 for histological analysis (blue circles). *mmp7*^-/- ^mice did not show clinical signs of EAE. Error bars represent SEM (*p < 0.05; **p < 0.005). (b) Haematoxylin and eosin staining of a brain section from a MOG-primed WT mouse shows perivascular cuffing of mononuclear cells. (c) Haematoxylin and eosin staining of a comparable section from a MOG-primed *mmp7*^-/- ^mouse shows no vascular cuffing. (d) Spinal cord sections from MOG-primed *wt *mouse showing cuffing (e) that was not seen in spinal cord sections from MOG-primed *mmp7*^-/- ^mice. (f) Spinal cord section from a MOG-primed *wt *mouse showing immune cells infiltration at the lateral edge and deeper into the parenchyma, (g) which was not seen in any spinal cord sections from similarly treated *mmp7*^-/- ^mice. Scale bars = 100 μm.

To determine if MMP-7 plays a critical role in EAE we compared the responses of *wt *and *mmp7*^-/- ^mice, congenic with the C57Bl/6 background, to MOG_35–55_. Clinical evidence of EAE was observed in 9/12 *wt *mice (75%), with scores up to 2.5 and an average peak near 1.6 (Figure 1a). Clinical scores showed paralysis beginning 12–14 days after MOG injection, which was consistent with previous reports of mice with this genetic background [42,43]. None of the *mmp7*^-/- ^mice showed any paralysis (0/9, 0%). Age-matched *wt *and *mmp7*^-/-^mice were injected simultaneously in 3 separate experiments, with similar results.

Haematoxylin and eosin staining of brain sections from *wt *mice isolated 15 days after MOG injection, near the peak of EAE, showed extensive mononuclear cell infiltration (Figure 1b, d). Perivascular cuffing, a classic feature of encephalitis, was not seen in the brains of MOG-primed *mmp7*^-/- ^mice (Figure 1c, e). *wt *mice also showed patches of demyelination adjacent to perivascular cuffs that were infiltrated by mononuclear cells and contained pycnotic nuclei (Figure 1f), all of which were absent in *mmp7*^-/- ^mice (Figure 1g). These findings indicate that *mmp7*^-/- ^mice are resistant to a stimulus that induces EAE in *wt *mice.

### Myeloid Cells Are the Primary Expressers of MMP-7 during EAE

**Figure 2:**
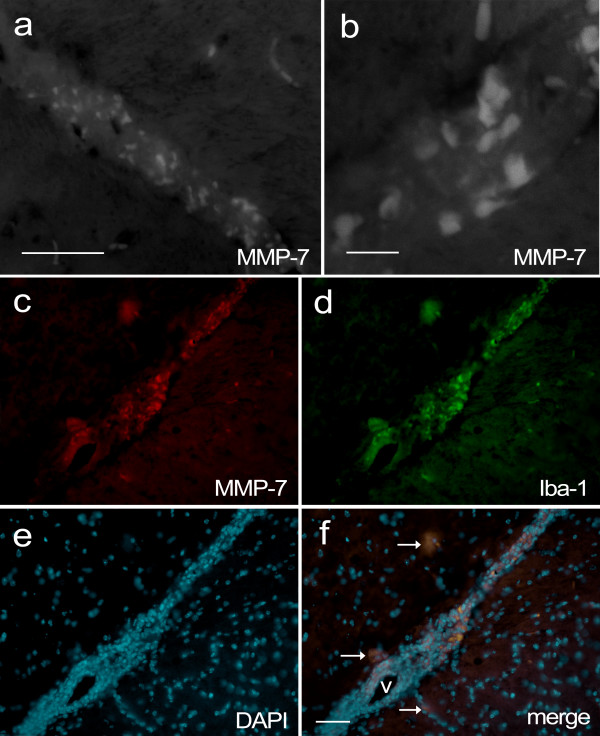
**Confocal and epifluorescence images of MMP-7 immuno-reactivity in the CNS of *wt *mice during EAE**. Brain sections were taken from a *wt *mouse 15 days after vaccination with MOG, when the EAE clinical score was 1. (a) Fluorescence microscope image of MMP-7 immunostaining in the brain of a *wt *mouse, scale bar = 50 μm. (b) Confocal microscope image of MMP-7 immunostaining in another region, scale bar = 10 μm. (c) MMP-7 immunostaining (red) shows strong labelling of many cells in the vascular cuff, (d) which co-localizes with Iba-1 immunoreactivity (green). (e) DAPI staining reveals the nuclear morphology of all cells in the cuff and surrounding parenchyma. (f) A merged image of c-e shows co-localization of Iba-1 and MMP-7 in the same cells. Arrows indicate double-stained cells in the parenchyma that have a spread-distended appearance. The lumen of the blood vessel is indicated with "v", scale bar = 100 μm.

MMP-7 expression in *wt *mice was localized by immunostaining sections from brains that were isolated during EAE, 15 days after vaccination. Previous reports have described elevated MMP-7 levels in brain and spinal cord during delayed type hypersensitivity and EAE, up to 500-fold over controls [11,13]. We observed strong MMP-7 immunostaining in perivascular cuffs (Figure 2a, b; see Additional file 1), in cells that co-stained for the monocyte marker Iba-1 (Figure 2c–f). Most MMP-7/Iba-1 immunopositive cells were in close proximity to vascular structures, but several cells were within the parenchyma and notably enlarged, possibly due to active phagocytosis. Further immunostaining revealed that MMP-7 also co-localized with tomato lectin and Gr-1 (see Additional file 2), common though less specific markers for myeloid cells. Although MMP-7 is an extracellular proteinase, it binds strongly to heparan sulphate on the surface of MMP-7-producing cells [44]. Interestingly, heparan sulfate proteoglycans have been reported to mediate monocyte migration across the BBB [45].

**Figure 3:**
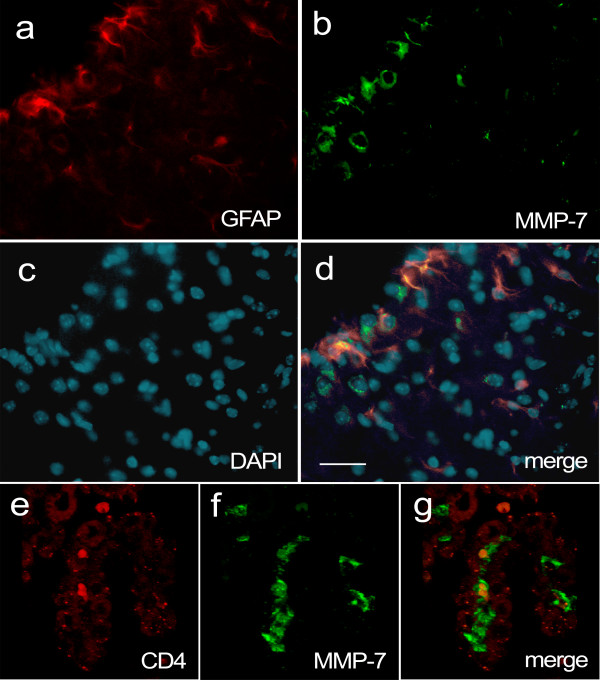
**MMP-7 immunoreactivity does not co-localize with the astrocyte marker GFAP, or CD4, in the CNS of *wt *mice during EAE**. Brain sections were taken from a *wt *mouse 15 days after vaccination with MOG, when the EAE clinical score was 1. (a) MMP-7 (green) and (b) GFAP (red) immunopositive cells. (c) DAPI staining of nuclei. (d) Merged image showing that GFAP and MMP-7 do not co-localize to the same cells although there are interactions between these two immunopositive populations. Scale bar = 50 μm. (e) CD4 immunostaining in the choroid plexus. Intensity of the red signal was increased to show choroid plexus structures, from auto-fluorescence. (f) MMP-7 immunostaining (green) shows several immunopositive cells between endothelial sheets, on the vascular side. (g) Merged image of e and f shows that CD4^+ ^and strong MMP-7 immunostaining do not co-localize. Arrow indicates continuity between an MMP-7 immunopositive cell and the lateral ventricle.

Endothelial cells in the cerebrovasculature are surrounded by astrocyte end-feet processes, which regulate BBB integrity. Reactive astrocytes produce a variety of factors in response to injury and infection, so we examined whether astrocytes expressed MMP-7 during EAE by co-immunostaining for glial fibrillary acidic protein (GFAP). Mice with high EAE clinical scores showed robust MMP-7 immunoreactivity around inflamed vascular structures, but within the parenchyma MMP-7 immunoreactivity did not co-localize with GFAP-positive cells (Figure 3a–d). Although astrocyte end-feet were adjacent to cells with strong MMP-7 staining, GFAP-positive cells did not appear to express the enzyme. These findings indicate that macrophages and not astrocytes are the primary producers of MMP-7 during EAE, which is consistent with other reports [46-48].

The choroid plexus consists of highly vascularized structures that produce cerebrospinal fluid (CSF) within brain ventricles. Although choroid plexus has highly fenestrated capillaries, blood components are kept separate from cerebrospinal fluid by tight junctions between specialized ependymal cells in the outer layer, constituting the blood-CSF barrier (BCB). Between fenestrated capillaries and ependymal cell layers is a basal lamina composed largely of collagen in which we observed strong immunoreactivity for MMP-7. Notably, CD4^+ ^T cells were observed between these layers but did not show MMP-7 immunoreactivity (Figure 3e–g). Accumulation of MMP-7 within the choroid plexus may degrade components of the basal lamina and connective tissue, or disrupt cadherin-mediated contacts. Indeed, confocal microscopy revealed patches of MMP-7 that appeared to breach the ependymal layer of the choroid plexus and become continuous with the CSF-containing lateral ventricle (Figure 3c). Significantly, such breaches in the BCB would permit the passage of immunoglobulins and serum proteins into the CSF, an important diagnostic indicator of MS.

### MMP-7-Deficiency Diminishes MOG-specific T cell Responses

To determine whether a lack of MMP-7 prevents MOG-specific immune responses, we assayed representative cell populations from *wt *and *mmp7*^-/- ^mice, *in vitro*. Splenocytes and lymphocytes were isolated from mice 15 days after MOG-injection and then cultured with MOG and ^3^H-thymidine. After 4 days, splenocytes from both *wt *and *mmp7*^-/- ^mice showed higher ^3^H-thymidine incorporation in response to MOG (10, 20, or 30 μg/ml) when compared with controls (see Additional file 3). However, MOG-induced proliferation of *mmp7*^-/- ^splenocytes was less than *wt *cells. Primary lymphocytes from MOG-primed *mmp7*^-/- ^mice also showed less proliferation in response to MOG *in vitro*, compared with *wt*. Higher density lymphocyte cultures showed similar results (not shown). These findings demonstrated that splenocytes and lymphocytes from *mmp7*^-/- ^mice have lower antigen-specific proliferation in response to MOG, *in vitro*.

**Figure 4:**
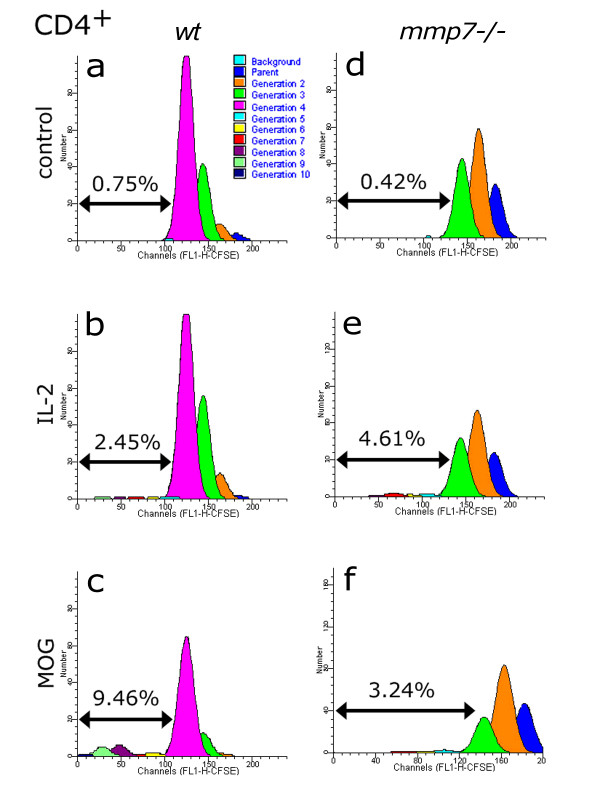
**Generation analysis of CD4^+ ^cells in mixed splenocyte/lymphocyte cultures isolated from MOG-primed *wt *and *mmp7*^-/- ^mice, using CFSE**. (a, b) CD4^+ ^cells from *wt *mice had a small response to IL-2, (c) and a more robust response to MOG (10 μg/ml). For a-c, percentages indicate the proportion of initial cells that divided beyond the 4^th ^generation, which was the farthest significant generation for media-only controls shown in a. (d, e) CD4^+ ^proliferation in response to IL-2 in *mmp7*^-/- ^mice was comparable to that in *wt *cells. (f) MOG-induced proliferation was comparable to that from IL-2 stimulation – less than the responses of *wt *cells to MOG.

The proliferation of T cells can only be inferred from ^3^H-thymidine incorporation studies, so we investigated MOG-specific responses of CD4^+ ^and CD8^+ ^T cells using CFSE pre-labelling and FACS analysis. After 4 days *in vitro*, control CD4^+ ^T cells from *wt *mice proliferated up to 4 generations, with further divisions making a negligible component (Figure 4a). Further proliferation was induced by IL-2 with a small, but measurable proportion of cells dividing up to 9 generations (Figure 4b). MOG induced a higher proportion of cells beyond 4 generations than did IL-2, constituting nearly 9.5% of the original cells that became CD4^+ ^(Figure 4c). CD4^+ ^T cells isolated from MOG-primed *mmp7*^-/- ^mice showed similar responses to IL-2, but less proliferation in response to MOG (Figure 4d–f). Although MOG-specific proliferation of CD4^+ ^T cells from MOG-primed *mmp7*^-/- ^mice was less with MOG-primed *wt*, MOG-specific proliferation was present.

**Figure 5:**
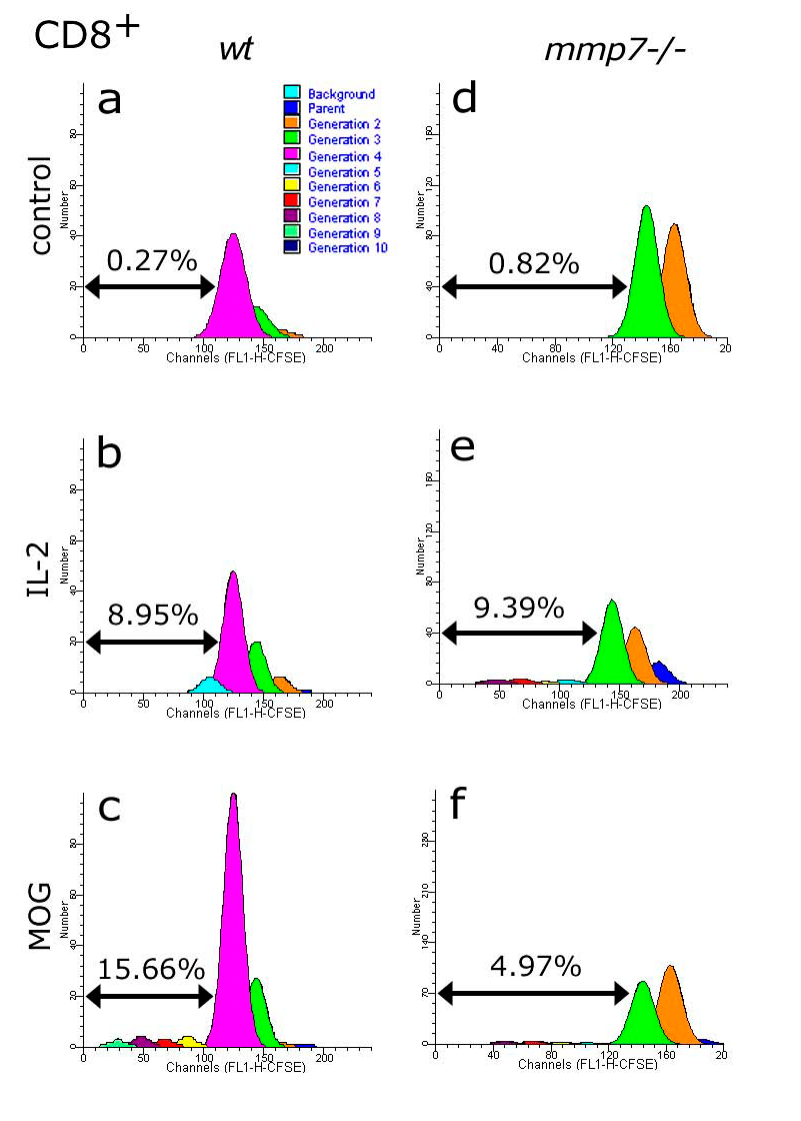
**Generation analysis of CD8^+ ^cells isolated from MOG-primed *wt *and *mmp7*^-/- ^mice, using CFSE**. (a, b) The proportion of IL-2-responsive progenitors that gave rise to CD8^+ ^cells in *wt *mice was comparable to that observed with (d, e) MMP-7^-/- ^mice. (c, f) MOG-responses were robust in *wt*, but not *mmp7*^-/- ^progenitors. Percentages indicate the proportion of progenitors that surpassed the farthest generation of media-only controls (a, d).

In addition to CD4^+ ^T cells, the transfer of myelin-specific CD8^+ ^T cells can also cause severe EAE [34]. CFSE analysis showed that CD8-expressing T cells did not proliferate past 4 generations in control cultures (Figure 5a). However, significant proliferation of up to the 10^th ^generation was observed in response to IL-2 (Figure 5b) and MOG (Figure 5c). CD8^+ ^T cells isolated from *mmp7*^-/- ^mice also showed minimal proliferation in control media (Figure 5d) and substantial proliferation in response to IL-2 (Figure 5e), but again MOG-induced proliferation was reduced compared with *wt *(Figure 5f).

### Transfer of T cells from MOG-primed Mice Induced EAE in wt but not *mmp7*^-/- ^Recipients

**Figure 6:**
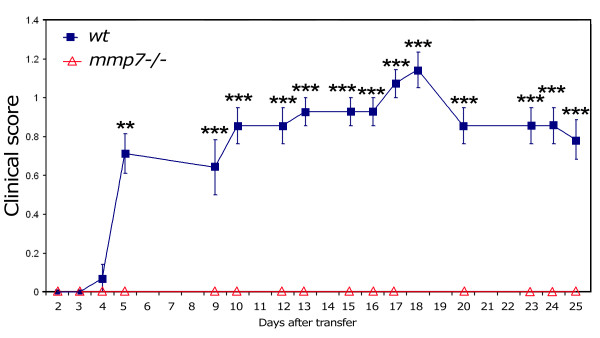
***mmp7*^-/- ^mice generate encephalitogenic T cells that produce EAE when transferred to *wt *recipients**. Graph indicating EAE scores in *wt *(n = 7) and *mmp7*^-/- ^(n = 6) recipients that received 20 × 10^6 ^re-stimulated splenocytes/lymphocytes from MOG-primed *mmp7*^-/- ^donors. Errors bars represent SEM (**p < 0.01; **p < 0.005).

We found that MOG-specific CD4^+ ^and CD8^+ ^T cell proliferation was reduced in *mmp7*^-/- ^mice, but still present, so we tested if MOG-specific T cells from *mmp7*^-/- ^mice were encephalitogenic using adoptive transfer. To examine this possibility we isolated splenocytes and lymphocytes from MOG-primed *mmp7*^-/- ^donors, re-stimulated with MOG *in vitro*, and then adoptively transferred those cells into naïve *mmp7*^-/- ^and *wt *recipients. Within 4 days all *wt *recipients (7/7) showed signs of paralysis that persisted for the entire 25 day post-injection period (Figure 6). Although EAE scores were low, difficulties with balance and gripping a tilted cage top were apparent in all *wt *recipients. In contrast, none of the *mmp7*^-/- ^recipients (0/6) showed any signs of EAE during the 25 day post-transfer period. These findings indicate that *mmp7*^-/- ^mice produce encephalitogenic T cells that can cause disease in *wt *mice, even though they have reduced T cell responses to MOG. In a reciprocal study, we found that splenocytes/lymphocytes from MOG-primed wt mice could also cause disease in wt mice (3/3), with clinical scores of 1–2, but *mmp7*^-/- ^recipients were resistant to the encephalitogenic effects of those cells (0/3). Therefore, *mmp7*^-/- ^mice are resistant to encephalitogenic T cells from *mmp7*^-/- ^or *wt *mice.

### MMP-7 Affects the Permeability of Brain Endothelial Cell Barriers

**Figure 7:**
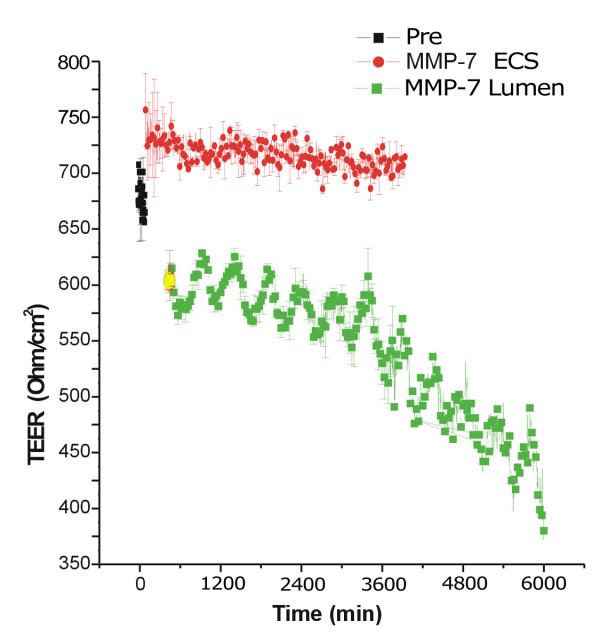
**Effects of MMP-7 on BBB permeability as measured in vitro by TEER**. Electrical potential between the extracellular side (ECS) and the luminal side as measured by TEER. After establishing the system, the TEER is typically ~650–700 Ohm/cm^2 ^(black). The addition of MMP-7 to the luminal side resulted in a 15–20% drop in TEER during a 2 h halt in flow (green). This drop in TEER continued over the next 4 days, eventually resulting in a 40% reduction in TEER. The addition of recombinant MMP-7 to the ECS (red) side did not result in a near or longer term decrease in TEER, and slightly increase TEER over the course of the experiment. These two graphs were made from different apparatus, run simultaneously.

To test whether MMP-7 affects tight junctions between cerebrovascular endothelial cells of the BBB, we used an established *in vitro *model consisting of porous hollow fibres and perfusion to replicate the pressure dynamics of brain capillaries [49]. Primary rat brain micro-vascular endothelial cells were grown inside the tubes and primary rat-brain astrocytes outside. Constant media flow through the lumen replicates the shear dynamics for vascular endothelial cells that line the tubes. After several days tight junctions connect endothelial cells lining the lumen to the extent that a trans-endothelial electrical resistance (TEER) can be measured. These junctions between endothelial cells depend on VE-cadherin, a substrate for MMP-7 [24]. We tested whether recombinant MMP-7 could alter permeability across these layers of brain endothelial cells by measuring the TEER. MMP-7 added to the brain-side (ECS) compartment did not decrease TEER and even slightly increased TEER by an as yet undetermined mechanism that may work through astroglia (Figure 7). However, MMP-7 affected the vascular endothelial cells when placed on lumenal side (Lumen) and caused a near-term 15–20% drop in TEER, with a subsequent drop of 40–45% after 4 days (Figure 7). This loss of electrical resistance is consistent with increased permeability of the endothelial cell layer lining the lumen of the micro-capillaries. Therefore, high local concentrations of MMP-7, as found on the surface of macrophages, can reduce the integrity of endothelial cell-to-cell contacts and may facilitate the transmigration of immune cells across the BBB.

## Discussion

High MMP-7 activity has been reported in demyelinating MS lesions and in the cerebrospinal fluid of MS patients [7-10,15], yet the role of this extracellular proteinase in MS is still unclear. Here, we have shown that *mmp7*^-/- ^mice are resistant to MOG-induced EAE. Diminished T cell responses to MOG make it less likely that EAE will develop, but *mmp7*^-/- ^mice still produce encephalitogenic T cells that can cause disease in *wt *mice. Immuno-localization of MMP-7 in areas adjacent to vascular structures suggests that it may facilitate immune access at the BBB. A common diagnostic feature of MS is MRI-detectable breaches of the BBB and spokes of demyelination that project outwardly from a cerebrovascular core (Dawson's fingers). Localization of MMP-7 to breaches of the BBB suggests that it may act directly on BBB integrity. We tested this possibility using an *in vitro *BBB model and found that the direct exposure of brain endothelial cells to recombinant MMP-7 increased the permeability between those cells. In contrast, MMP-7 did not increase permeability on the brain side of the BBB, but it may have other effects.

Inside the BBB, MMP-7 may contribute to EAE by activating MMP-2 and MMP-9 (proform), or by reducing immune privilege by cleaving FasL [50]. As a potent inducer of apoptosis in activated T cells, FasL is crucial to the recovery phase of EAE. Mice with defective FasL undergo more severe and prolonged EAE, compared with WT mice [51]. Indeed, CNS injury models have shown that a localized increase of FasL expression within the CNS is a consistent response to breaches of the BBB [33,34]. The potent activity of FasL can be attenuated by shedding the Fas-binding domain by MMP-7 [28,38,39]. Macrophages that infiltrate the CNS and produce high local concentrations of MMP-7 may cause localized shedding of FasL and create discrete pockets of lowered immune privilege within the CNS. This process could prolong the survival of activated T cells that would otherwise die by activation induced cell death or FasL-induced apoptosis. Interestingly, granulocyte macrophage colony stimulating factor (GM-CSF) knockout mice are also resistant to MOG-induced EAE [52]; however, their resistance is attributed to attenuated MOG-specific T cell responses, perhaps due to fewer antigen-presenting cells. Variables that affect macrophage availability might be expected to have a direct impact on MMP-7 production near cerebrovascular structures and also affect EAE susceptibility.

Within the brain MMP-7 may also contribute to memory, motor and cognitive problems that often accompany MS episodes. Recent studies have shown that MMP-7 and MMP-9 can disrupt mature dendritic spines, causing them to assume immature morphologies, which greatly reduces the synaptic strength of excitatory synapses and can impact memory and behaviour [53,54]. Furthermore, EphB receptors are critically important for the formation and maintenance of mature dendritic spines, and MMP-9 has recently been shown to cleave EphB [55]. Infiltrating macrophages in perivascular cuffs or near demyelinating lesions might produce enough MMP-7 to activate extracellular pools of MMP-9 (proform) and disrupt synapse stability in nearby areas – even without demyelination or overt pathology [56].

Several cytokines, including TNF-α, have been shown to increase MMP-7 expression and have been repeatedly linked with EAE and MS inflammation [57,58]. IFN-β is a promising treatment for MS and has been shown to repress MMP-7 and MMP-9 expression for patients with relapsing-remitting MS [18,51]. Our findings suggest that MMP-7 plays a role in the extravasation of immune cells during EAE. MMP-7 expression adjacent to the cerebrovasculature and ependymal cells of the choroid plexus suggests that metalloproteinase inhibitors could prevent EAE by blocking MMP-7 activity at these sites. Interestingly, hydroxamate-based metalloproteinase inhibitors can prevent EAE, but may not be able to cross an intact BBB, so their ability to prevent EAE would depend on actions after the BBB has been compromised unless they work by inhibiting MMPs outside of the BBB, such as MMP-7 on circulating monocytes [29].

## Conclusion

This study demonstrates that MMP-7 plays a critical role in MOG-induced EAE in C57Bl/6 mice, although it is possible that stronger encephalitogenic stimuli (e.g. myelin basic protein) in other mouse strains may respond differently to MMP-7 deficiency. The reduced response of CD4^+ ^or CD8^+ ^T cells to EAE, did not preclude the production of encephalitogenic T cells, and may reflect an important role for MMP-7 in the antigen-presentation or the specialized microenvironment of lymphoid organs. Immuno-localization of MMP-7 to myeloid cells and cerebrovascular structures, along with its ability to increase the permeability between endothelial cells, implicates this enzyme in compromising BBB and/or BCB integrity, and suggests that MMP-7 may have additional effects in the brain parenchyma that extend beyond lymphocyte infiltration.

## Methods

### Induction of EAE

The generation and characterization of *mmp7*^-/- ^mice on the C57BL/6 background has been reported [59]. Mice that had been back-crossed for at least 10 generations, onto the C57Bl/6 background, were bred in the University of California, Riverside (UCR) vivarium in accordance with Institutional and NIH animal care and use guidelines. Age-matched *mmp7*^-/- ^and wt (B6) mice were injected at 7–18 weeks of age with 100 μl of CFA emulsion containing 250 μg of recombinant MOG_35–55_, divided into three sites on shaved backs. Recombinant MOG was synthesized at the University of California Los Angeles (UCLA) peptide core and was 97–99% pure. One and 3 days after MOG injections, mice were given intra-peritoneal injections of 200 ng pertussis toxin each. Each day, mice were scored for clinical signs of EAE as follows: 0 = no EAE; 1 = total loss of tail tonicity; 2 = hind limb weakness, impaired righting reflex, or forelimb impairment alone; 3 = total paralysis of one or both hind limbs; 4 = hind and forelimb paralysis; 5 = moribund, death [60]. Statistical analysis was done by paired Student-t Tests (2-tailed) of *wt *and *mmp7*^-/- ^clinical scores at each time-point.

### Splenocyte and Lymphocyte Isolation

Spleen and draining lymph nodes were isolated from experimental and control mice 15–24 days after post MOG injection. Tissues were disrupted in complete RPMI with 5–7 strokes of a glass Dounce (15 mL Pyrex). Single-cell suspensions were prepared by passing homogenates over a 70-μm sieve filter (BD, Franklin Lakes, NJ) and rinsing with 2 mL of complete RPMI. Cells were pelleted and red blood cells lysed by resuspension in cold buffer (Red blood cell lysis buffer, Sigma, St. Louis, MO) for 5 min on ice. Next, cells were pelleted and resuspended in RPMI 1640 media containing 10% FCS (Hyclone, Logan, UT), L-glutamine, sodium bicarbonate, sodium pyruvate, essential and non-essential amino acids, vitamins, penicillin/streptomycin, and β-mercaptoethanol (complete RPMI), and maintained in a humidified CO_2 _incubator at 37°C. Viable cells were counted by trypan blue exclusion.

### ^3^H-Thymidine Measurement of Proliferation

Cells were isolated from 3 *wt *and 3 *mmp7*^-/- ^mice 15 days after vaccination with MOG, as described. Quadruplicate wells of each condition consisted of 2 or 4 × 10^5 ^cells/well in a 96-well plate, and cultured in the presence of 0, 10, 20 or 30 μg/mL MOG_35–55 _overnight at 37°C. Cells were then pulsed with 1 μCi/well of ^3^H-thymidine (ICN Pharmaceuticals, Costa Mesa, CA) for the last 4 h of culture, and processed using a cell harvester (PHD, Cambridge Technologies). Each sample was cut from the filter and radioactivity measured with a scintillation counter. Mean values from quadruplicates were used to generate proliferation responses that were normalized to the control condition (0 μg/mL MOG) for cells from each animal. These normalized proliferation responses were averaged for all mice of each genotype.

### CFSE Labelling and Analysis

Freshly isolated splenocytes and lymphocytes were pooled, pelleted, and then resuspended in PBS at 6 × 10^7 ^cells/ml. An equal volume of PBS containing 10 μM carboxy-fluorescein diacetate succinimidyl ester (CFSE; Molecular Probes, Inc. Eugene, OR) was added, then cells were gently mixed and incubated for 20 min at 37°C. Cells were pelleted and washed twice with PBS. Immediate FACS analysis of an aliquot determined cell labelling efficiency, typically >99%, and those values were used as baseline labelling of the parental generation. CFSE-labelled cells were then resuspended (2.5 × 10^6 ^cells/ml) in complete RPMI with 0, 10, or 15 μM MOG, or 25 units/ml IL-2 and incubated at 37°C for 4 days. Aliquots of each preparation were then labelled with fluorescently-conjugated (PE) anti-CD4, anti-CD8, or isotype-specific controls (BD Biosciences, San Diego, CA) at 1 μg/10^6 ^cells, 25 min at 4°C. Cells were then rinsed with PBS (3×) and fixed with an equal volume of 2% paraformaldehyde. Data was acquired using a FACScan^® ^(Becton Dickinson, San Jose, CA), and analyzed using ModFit™ software proliferation wizard (Becton Dickinson, San Jose, CA). Typically, 15–50,000 events were collected for each sample.

### Adoptive Transfer

Primary splenocytes and lymphocytes were isolated from MOG-primed *mmp7*^-/- ^mice 10 days after vaccination and cultured with 10 μg/ml MOG and 10 ng/ml IL-2 (2.5 × 10^6 ^cells/ml) for 5 days. Recipient mice received 20 × 10^6 ^cells in 0.2 mL of PBS by tail-vein injection.

### Immunostaining

Immediately following the removal of spleen and lymph nodes each mouse was perfused (cardiac) with saline containing heparin, saline alone, and then fresh 4% paraformaldehyde in PBS. The isolated cranium and spinal column were post-fixed overnight in paraformaldehyde, after which the brain and spinal cord were carefully removed. Tissue was cryo-protected with increasing sucrose concentrations and then sectioned on a cryostat (7 μm), and stored at 4°C until use. Slide-mounted sections were warmed to 37°C for 10 min, rinsed with PBS, and non-specific antigens blocked with either 10% BSA or 10% normal goat serum (NGS) for 1 h, depending on the sources and specificities of the antibodies to be used. Antibodies used were: mouse anti-MMP-7 (1:100; Dr. Carole Wilson), rabbit anti-MMP-7 (1:100; Dr. Carole Wilson), mouse anti-CD4-PE (1:100; BD Biosciences), mouse anti-GFAP-PE (1:2000; Sigma), mouse anti-Gr-1 (BD Biosciences), mouse Iba-1-FITC (1:100; Genetex, #1022-5). Secondary antibodies used were FITC-conjugated anti-rabbit IgG (1:100; Sigma) and Cy3-conjugated anti-rat IgG (1:100; Molecular Probes); Alexa-488 conjugated anti-mouse (1:1000; Invitrogen).

### *In vitro *BBB Model

The system used has been described in detail previously [61,62]. Briefly, primary rat-brain micro-vascular endothelial cells were cultured on the lumen surface of porous tubes, and grown with constant perfusion of media through the lumen of the tubes. Outside the tubes were grown primary rat astrocytes, which supplied signalling molecules necessary to create tight junctions between endothelial cells sufficient to be recorded as resistance. Experiments were done at week 3 of EC-astrocyte co-culture in this dynamic in vitro BBB model when all tubes were measured for resistance and only those showing significant resistance were used. Active recombinant human MMP-7 (Millipore, Temecula, CA) was added to the extra capillary space (astrocyte) side of the system at 1 or 5 units per mL (1^st ^group of experiments), or to the lumenal (endothelial) side at 1 unit per mL (2^nd ^group of experiments). In both experimental series MMP-7 was added with continuous perfusion in one set of experiments, or with a 1.5 h pause of flow followed by reperfusion in separate experiments. Permeability of the endothelial sheets was measured by standard electrophysiological recording as the trans-endothelial electrical resistance (TEER) between the ECS and lumen.

## Abbreviations

BBB: blood-brain barrier; BCB: blood-CSF-barrier; CFSE: carboxy-fluorescein diacetate succinimidyl ester; CNS: central nervous system; CSF: cerebrospinal fluid; EAE: experimental autoimmune encephalomyelitis; ECM: extracellular matrix; FasL: Fas ligand; GFAP: glial fibrillary acidic protein; GM-CSF: granulocyte macrophage colony stimulating factor; MMP: matrix metalloproteinase; *mmp7*^-/-^: MMP-7 deficient; MOG: myelin oligodendrocyte glycoprotein; MS: multiple sclerosis; NGS: normal goat serum; sFasL: soluble Fas ligand; TEER: trans-endothelial electrical resistance; UCR: University of California Riverside; *wt*: wild-type.

## Authors' contributions

LAB and RS performed adoptive transfers and evaluated clinical scores. DWE designed and supervised the study, performed immunostaining, and wrote the manuscript. EG assisted with immunological assays. CLW provided *mmp7*^-/- ^mice and MMP-7 specific antibodies. LK-B and DJ performed in vitro BBB assays. All authors read and approved the final manuscript.

## Supplementary Material

Additional file 1 **Movie from a confocal image stack showing MMP-7 immunopositive cells in a perivascular cuff from a MOG-primed *wt *mouse, during EAE.** The mouse had been vaccinated with MOG 15 days prior and demonstrated a clinical score of 1 at the time of sacrifice. Click here for file

Additional file 2 **Confocal microscopy shows co-localization of MMP-7 with tomato lectin and Gr-1.** (a) Confocal image of tomato lectin-stained cells (red) in a vascular cuff in the brain of a *wt *mouse during EAE, 15 days after vaccination with a clinical score of 1. (b) Confocal image of the same section in a showing MMP-7 immunostaining (green). (c) Merged image of boxes a and b shows co-localization of MMP-7 and tomato lectin in a perivascular accumulation of immune cells during EAE. (d) Confocal image of Gr-1 immunopositive cells (red) in the brain of a WT mouse during EAE. (e) Confocal image of the same section in d showing MMP-7 immunopositive cells (green). (f) Merged image of d and e shows co-localization of MMP-7 and Gr-1. Click here for file

Additional file 3 **Proliferation responses of splenocytes and lymphocytes isolated from MOG-primed *wt *and *mmp7*^-/- ^mice. **(a) ^3^H-Thymidine incorporation of splenocytes isolated from MOG-primed *wt *and *mmp7*^-/- ^mice and re-stimulated for 4 days in vitro with 0, 10, 20, or 30 μg/ml MOG. (b) ^3^H-Thymidine incorporation of lymphocytes from the same mice and re-stimulated for 4 days in vitro with 0, 10, 20 or 30 μg/ml MOG. Although proliferation tended to be lower in splenocytes and lymphocytes isolated from *mmp7*^-/- ^mice, the differences were not statistically significant in comparison to cells from *wt *mice. Click here for file
