# Supplementary figures and images for: Matrix metalloproteinase-7 facilitates immune access to the CNS in experimental autoimmune encephalomyelitis
Source: BMC Neurosci. 2009 Mar 6;10:17. doi: 10.1186/1471-2202-10-17 (PMC2660336; doi:10.1186/1471-2202-10-17)

Supplemental Figure 2

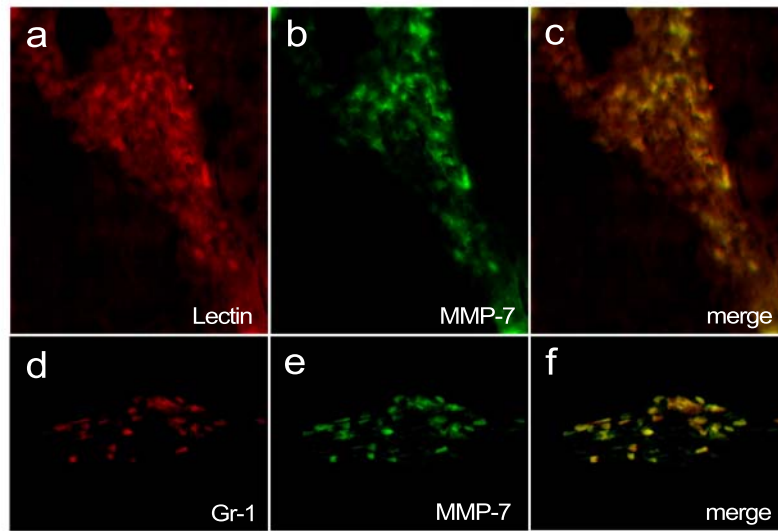

Supplement: Additional file 2 — Confocal microscopy shows co-localization of MMP-7 with tomato lectin and Gr-1. (a) Confocal image of tomato lectin-stained cells (red) in a vascular cuff in the brain of a wt mouse during EAE, 15 days after vaccination with a clinical score of 1. (b) Confocal image of the same section in a showing MMP-7 immunostaining (green). (c) Merged image of boxes a and b shows co-localization of MMP-7 and tomato lectin in a perivascular accumulation of immune cells during EAE. (d) Confocal image of Gr-1 immunopositive cells (red) in the brain of a WT mouse during EAE. (e) Confocal image of the same section in d showing MMP-7 immunopositive cells (green). (f) Merged image of d and e shows co-localization of MMP-7 and Gr-1. [file 1471-2202-10-17-S2.pdf]

Supplemental Figure 3

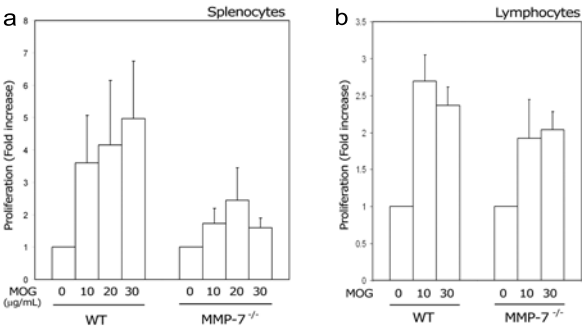

Supplement: Additional file 3 — Proliferation responses of splenocytes and lymphocytes isolated from MOG-primed wt and mmp7-/- mice. (a) 3H-Thymidine incorporation of splenocytes isolated from MOG-primed wt and mmp7-/- mice and re-stimulated for 4 days in vitro with 0, 10, 20, or 30 μg/ml MOG. (b) 3H-Thymidine incorporation of lymphocytes from the same mice and re-stimulated for 4 days in vitro with 0, 10, 20 or 30 μg/ml MOG. Although proliferation tended to be lower in splenocytes and lymphocytes isolated from mmp7-/- mice, the differences were not statistically significant in comparison to cells from wt mice. [file 1471-2202-10-17-S3.pdf]
